# Inversion-based genomic signatures

**DOI:** 10.1186/1471-2105-10-S1-S7

**Published:** 2009-01-30

**Authors:** Krister M Swenson, Bernard ME Moret

**Affiliations:** 1 Laboratory for Computational Biology and Bioinformatics, EPFL (Swiss Federal Institute of Technology), EPFL-IC-LCBB, INJ 230, Station 14, CH-1014 Lausanne, Switzerland

## Abstract

**Background:**

Reconstructing complete ancestral genomes (at least in terms of their gene inventory and arrangement) is attracting much interest due to the rapidly increasing availability of whole genome sequences. While modest successes have been reported for mammalian and even vertebrate genomes, more divergent groups continue to pose a stiff challenge, mostly because current models of genomic evolution support too many choices.

**Results:**

We describe a novel type of genomic signature based on rearrangements that characterizes evolutionary changes that must be common to all minimal rearrangement scenarios; by focusing on global patterns of rearrangements, such signatures bypass individual variations and sharply restrict the search space. We present the results of extensive simulation studies demonstrating that these signatures can be used to reconstruct accurate ancestral genomes and phylogenies even for widely divergent collections.

**Conclusion:**

Focusing on genome triples rather than genomes pairs unleashes the full power of evolutionary analysis. Our genomic signature captures shared evolutionary events and thus can form the basis of a robust analysis and reconstruction of evolutionary history.

## Background

### Introduction

The study of evolution is a study of patterns of change, but also of conservation, the latter being typically easier to detect and characterize. Moreover, elements conserved across many species were probably present in their last common ancestor and preserved through selection pressures, so that these conserved elements probably play a major role in the fitness of the organisms. Biologists have long studied patterns of conservation in DNA sequences: first pairwise sequence similarity in large databases (as in the widely used FASTA [1] and BLAST [2]), then multiple sequence alignments and phylogenetic reconstruction, and finally the reconstruction of ancestral sequences, an avenue of enquiry that has seen much activity of late (see, e.g., [3]). Recently, researchers have also started to look for characteristic patterns of change across a collection of species–an example being the *discriminating subsequences *of Angelov *et al*. [4].

All of these efforts aim at recovering what one could term *genomic signatures*–subsequences that best characterize the evolutionary history of the given group of organisms. The original use of the term "genomic signature" referred to the spectrum of dinucleotide frequencies gathered from the entire genome of organelles and of some prokaryotes [5,6]. Since then, it has been used for genome-wide gene expression data [7,8], protein-based (or gene-family-based) comparisons [9], genome-wide localization of transcription factors (so-called genomic signature tags) [10], and many other variations. These uses all share a genome-wide scope and a particular technique for capturing conservation and/or divergence in the genome. Recovering such signatures would enhance our understanding of genomic evolution as well as provide an important tool in biomedical research.

The focus to date in evolutionary genomics has been on DNA sequence evolution, in part because of the nature of the available data (collections of gene sequences form the overwhelming majority of biomolecular data) and in part because of their relative simplicity. The assumed model of evolution has been a simple process of point mutation and gap-forming indels. However, other processes affect the evolution of a genome, including large-scale events that rearrange genes along the chromosomes, introduce new genes, or remove existing ones. Rearrangements, in particular, interfere with our ability to align sequences: for instance, a single *inversion *(in which a segment of genes is reversed in place) can make two sequences unalignable under the mutation and indels model.

As more and more genomes are fully sequenced, interest in reconstructing complete ancestral genomes has grown; Pevzner's group, for instance, has published extensively on the topic in the context of vertebrate genomes (see, e.g., [11,12]), as has a group headed by Haussler and Miller [13]. However, while rearrangements such as inversions, transpositions, translocations, and others are complex and powerful operations, our models for them remain poorly parameterized, often reduced to the simplest case of uniform distributions. Under such models, reconstruction of ancestral genomes for organisms that exhibit significant divergence (in contrast to mammals or even vertebrates) remains poor, mostly due to the enormous number of equally "good" evolutionary scenarios [14]. It is therefore natural to turn once again to genomic signatures, this time formulated in terms of a rearrangement (rather than a sequence evolution) model.

In this paper we introduce a measure of similarity defined between two genomes *with respect to a third*. The key idea is the introduction of the third genome, which allows us to take into consideration the evolutionary paths from the two genomes under study to the third, thus basing our measure of similarity on the evolutionary history of the two genomes rather than just on their current configuration. Naturally, these evolutionary paths are not unique under current models and thus a number of ancestral states can be reached on the way from the two genomes under study to the third genome. We call these states *rearrangement signatures *and further distinguish those that are farthest from the third genome (the most recent, as viewed from the perspective of our two genomes under study) as *maximum rearrangement signatures*. Although the concepts introduced here apply to any rearrangement operation, we study these signatures under the operation of inversion, the most commonly used rearrangement operation in work to date [15]. We show that maximum signatures carry much information about ancestral genomes and that they can often be computed within a reasonable amount of time in spite of the very large search space. We use simulations under a wide variety of conditions to show that the maximum signatures pinpoint the true ancestral genome, either recovering it outright or producing one very close to it, and to show that these signatures can be used to reconstruct reliable phylogenies, all using a polynomial-time heuristic that runs much faster than a full exhaustive search.

### Notation and definitions

**Figure 1:**
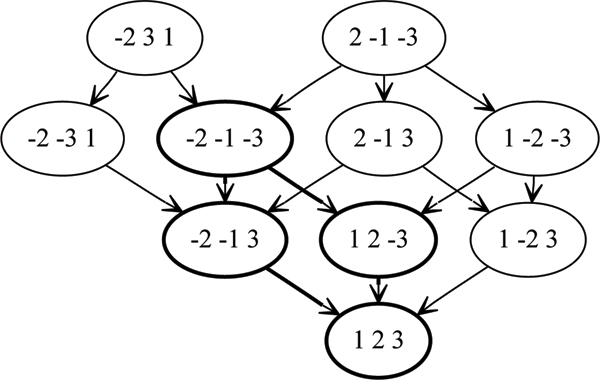
The union of the inversion lattices for *π*_*A *_= {-2 3 1, *π*_*B *_= 2 -1 -3}, and *π*_*L *_= 1 2 3. The signature graph is highlighted in bold.

As is usual in the study of rearrangements, we represent a chromosome of *n *genes by a signed permutation on the elements {1, 2, ..., *n*}. Given a signed permutation *π*, an *inversion r*(*i, j*) is a permutation that, when applied to *π*, reverses the order and the sign of a segment of *π *that begins at the *i*th gene and ends at the *j*th one. Thus, if we write the identity permutation as 1, ..., *i *- 1, **i**, **i **+ **1**, ..., **j **- **1**, **j**, *j *+ 1, ..., *n*, then *r*(*i, j*) becomes 1, ..., *i *- 1, -**j**, -(**j **- **1**), ..., -(**i **+ **1**), -**i**, *j *+ 1, ..., *n*. *r π *denotes the application of inversion *r *to permutation *π*. For signed permutations *π *and *π'*, the *(inversion) edit distance d *(*π*, *π'*) is the minimum number of inversions needed to transform *π *into *π'*. We say that a sequence of permutations *π*_0_, *π*_1_, ..., *π*_*d *_forms an *edit path *if for all *π*_*i*_, 0 ≤ *i *<*d*, we have *d*(*π*_*i*_, *π*_*i *+ 1_) = 1; each inversion applied along this path is then deemed an *edit inversion*. Taking each *π*_*i *_to be a vertex and linking two vertices with an edge whenever the corresponding permutations occur consecutively on an edit path creates an *edit path graph*. The relation "is on the edit path from" thus induces a partial order, the *edit partial order*, or *EPO*. We denote the EPO between *π*_0 _and *π*_*d *_as $EPO_{\pi_{0}}$ (*π*_*d*_) or $EPO_{\pi_{d}}$ (*π*_0_). So if we have *π*_3 _= 2 -1 -3 and *π*_0 _= 1 2 3 then an edit path between them might visit permutations *π*_2 _= -2 -1 -3 and *π*_1 _= -2 -1 3 before reaching *π*_0_. Figure 1 shows the EPOs for 2 -1 -3 and -2 3 1.

We are interested in the intersection of EPOs, which will yield the desired inversion signatures. For a set of *k + *1 permutations, one of which is the reference permutation called the *locus*, an *inversion signature *is the permutation corresponding to a vertex in the intersection of the *k *EPOs from each of the other *k *permutations to the locus.

**Definition 1 ***The set of all *inversion signatures *for permutations π*_1_, ..., *π*_*k*_*with locus π*_*L *_*is *$S_{\pi_{L}}(\pi_{1},...,\pi_{k})=V\left(EPO_{\pi_{L}}(\pi_{1})\cap EPO_{\pi_{L}}(\pi_{2})\cap \cdots \cap EPO_{\pi_{L}}(\pi_{k})\right)$, *where V*(*G*) *denotes the set of vertices of graph G*.

Whenever the context is unambiguous, we shall simply write $S_{\pi_{L}}$ for $S_{\pi_{L}}$ (*π*_1_, ..., *π*_*k*_). Similarly, the *signature graph *on *π*_1_, ..., *π*_*k *_with respect to *π*_*L *_is the graph $EPO_{\pi_{L}}(\pi_{1})\cap EPO_{\pi_{L}}(\pi_{2})\cap \cdots \cap EPO_{\pi_{L}}(\pi_{k})$. An inversion signature *π*_*s *_∈ $S_{\pi_{L}}$ is thus a permutation that embodies some of the commonality between the *k *other permutations with respect to *π*_*L*_, in the sense that they all possess an edit path to *π*_*L *_that passes through *π*_*s*_. A *maximum signature *is a signature in $S_{\pi_{L}}$ that is as far away from *π*_*L *_(and thus as close to the *k *other permutations) as possible.

**Definition 2 ***The set of all *maximum signatures *is *$S_{\pi_{L}}^{*}=\{\pi_{s}\in S_{\pi_{L}}|for\;all\;{\pi^{\prime }}_{s}\in S_{\pi_{L}},d(\pi_{L},\pi_{s})\geq d(\pi_{L},{\pi^{\prime }}_{s})\}$.

A maximum inversion signature is thus a permutation that represents the "maximum commonality" between the *k *permutations: it is as close to these *k *permutations as possible while still being part of all edit paths to *π*_*L*_. From a biological perspective, this edit path from *π*_*L *_to the signature can be thought of as the evolution that happened before speciation, or the pattern of change that the *k *sequences have in common.

As with the special case for Steiner points called the *median *[16], we find it helpful to name the case with *k + *1 = 3. For this case we have two permutations *π*_*A *_and *π*_*B *_and an ancestor locus *π*_*L *_and we call $S_{\pi_{L}}^{*}$ (*π*_*A*_, *π*_*B*_) the *pairwise maximum signature*.

In Figure 1 we have *π*_*A *_= 2 -1 -3, *π*_*B *_= -2 3 1, and *π*_*L *_= 1 2 3 (the *identity *permutation of length 3). The signature graph is outlined in bold. The signatures in this case are -2 -1 -3 -2 -1 3, 1 2 -3, and the trivial signature *π*_*L *_= 1 2 3. The only maximum signature is also the only maximal signature -2 -1 -3.

## Methods

We begin with an investigation of rearrangement-based genomic signatures as defined above, then give procedures for signature-based phylogenetic and ancestral reconstruction.

### Computing signatures

Definition 1 can be restated inductively in terms of edit paths that move from the locus *π*_*L *_towards the other permutations *π*_1_, ..., *π*_*k*_. We say that some permutation *π *has a *common edit inversion r *with respect to *π*_1_, ..., *π*_*k *_if we observe *d*(*π*_*L*_, *π*_*i*_) - *d*(*π*_*L*_, *rπ*_*i*_) = 1 for 1 ≤ *i *≤ *k*.

**Definition 3 ***The locus π*_*L*_*is an inversion signature for permutations π*_1_, ..., *π*_*k*_. *If permutation π is an inversion signature and r is a common edit inversion with respect to π*_1_, ..., *π*_*k*_, *then rπ is also an inversion signature*.

Thus, starting at the locus (which is the smallest possible signature), one can enumerate all signatures by repeatedly applying every possible common edit inversion to the current collection of signatures; maximal signatures are those signatures for which no common edit inversion exists and maximum signatures are the largest of these maximal signatures (i.e., the farthest away from the locus). Common edit inversions form the basis for the MGR algorithm of Bourque and Pevzner [12], who used a greedy algorithm that picks a single path by always choosing the common edit inversion that provides the largest number of common edit inversions at the next step.

**Figure 2:**
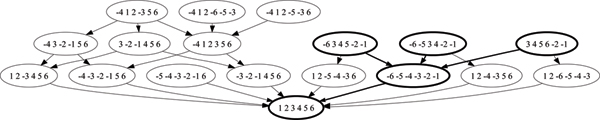
The signature graph for *π*_*A *_= -4, 1, -5,2, -6, 3, *π*_*B *_= -4, 1, 6, 2, -5, 3, and *π*_*L *_= 1, 2, 3, 4, 5, 6.

The signature space is of course very large. In particular, if the two permutations of interest are just one inversion apart, then the space of all signatures can be roughly the same size as the inversion EPO between one of the permutations and the locus–and that is, in expectation, exponentially large in the pairwise distance. (However, the complexity of finding a maximal signature is unknown at this time.) We use the greedy heuristic of MGR to construct maximal signatures and show that it often returns the maximum signature. It is not optimal, however: consider the permutations *π*_*A *_= -4 1 -5 2 -6 3, *π*_*B *_= 4 1 6 2 -5 3, and *π*_*L *_= 1 2 3 4 5 6. In the signature graph of Figure 2, vertices that can be produced by the greedy heuristic are highlighted, none of which are a maximum signature.

### Noninterfering independent sets

We say that a set of edit inversions on a permutation *π commutes *iff applying every inversion in the set always yields the same permutation *τ*, regardless of the order in which the inversions are applied. (Trivially, inversions that operate on disjoint intervals commute.)

**Definition 4 ***A set of n inversions on π with respect to τ is *noninterfering *if and only if it commutes and applying these inversions in any order reduces by n the inversion distance between π and τ*.

Commuting and noninterfering inversions offer a way to reduce the search space in computing a median or in examining all sorting paths: for a set of size *n*, it is enough to look at a single ordering of its inversions rather than at all *n*! possible orderings [17].

The concept of noninterfering inversions extends naturally to our framework with a defined ancestor.

**Definition 5 ***A set of inversions R is *mutually noninterfering *for π*_*A *_*and π*_*B *_*with locus π*_*L *_*if it is noninterfering for π*_*L *_*with respect to π*_*A *_*and also for π*_*L *_*with respect to π*_*B*_.

Such mutually noninterfering sets form the basis for another greedy algorithm: we repeatedly find and apply to *π*_*L *_sets of mutually noninterfering inversions until there are none left. Mutually noninterfering sets can be found very quickly, so a greedy algorithm based on this approach runs very fast. We use this particular greedy heuristic in our experiments.

### Signature-based tree reconstruction

Since signatures are just nodes along evolutionary paths, they can be used as internal nodes in a process of phylogenetic reconstruction. We begin with a naïve algorithm to illustrate the basic approach.

The idea is to overlay the EPOs from each of the leaves *π*_1_, ..., *π*_*k *_to the locus *π*_*L *_and construct a tree representative of the resulting structure. Consider the set of these EPOs, *O *= {$EPO_{\pi_{L}}$(*π*_*i*_)| 1 ≤ *i *≤ *k*}; our algorithm constructs a tree from the current version of *O*, iteratively choosing a node from pairwise intersections of graphs in *O *and updating *O *to reflect this choice. Specifically, at iteration *i*,

1. select from *O *a vertex *π*_*s *_that maximizes *d *(*π*_*L*_, *π*_*s*_);

2. if the vertex selected in the previous step belongs to the intersections of *P*_*A*_, *P*_*B *_∈ *O*, then create a node in the tree to be the parent of the subtrees represented by *P*_*A *_and *P*_*B*_;

3. in *O *replace $EPO_{\pi_{L}}$ (*π*_*A*_) and $EPO_{\pi_{L}}$ (*π*_*B*_) with their intersection.

This algorithm yields a tree without internal node labels, because EPOs are not closed under intersection, so that a node in the tree may represent two graphs from *O *that no longer have a least upper bound.

Our second algorithm overcomes this problem; in addition, it yields implicit edit paths from the leaves to the root that join at the internal nodes. In this improved version, we maintain the invariant that elements of *O *are always EPOs. Thus only the third step of the iteration is affected, and replaced by the following:

• in *O *replace $EPO_{\pi_{L}}$ (*π*_*A*_) and $EPO_{\pi_{L}}$ (*π*_*B*_) with $EPO_{\pi_{L}}$ (*π*_*s*_).

Step 1 in each iteration is obviously the computationally intensive one; our implementation for this step uses the MGR heuristic.

### Distance-based bound on signature size

**Figure 3:**
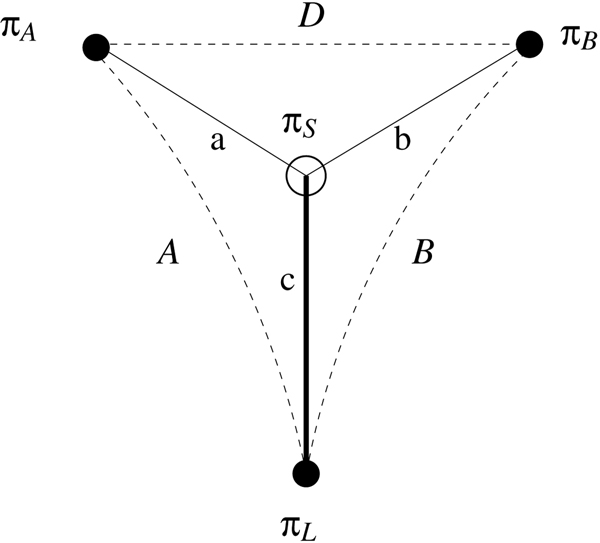
The distances around a signature *π*_*S*_.

We develop an upper bound based on pairwise distances to help us evaluate our greedy signature methods in the experimental phase. Denote by *A*, resp. *B*, the inversion distance between the locus and *π*_*A*_, resp. *π*_*B*_, and by *D *the inversion distance between *π*_*A *_and *π*_*B*_. (Inversions distances can be computed in linear time [18].) Now consider some arbitrary signature *π*_*S *_for this triple and denote its size, or distance from the locus, by *c*; Figure 3 depicts the situation. As all distances are edit distances, we can write *A *- *a *= *B *- *b *and, by the triangle inequality, *a *+ *b *≥ *D*; combining the two, we get

$$a\geq \frac{D+A-B}{2},$$

with the symmetric version for *b*. Without loss of generality, assume *A *≥ *B*; then we get

$$d(\pi_{L},\pi_{S})c\leq A-\left(\frac{D+A-B}{2}\right),$$

the desired upper bound.

## Results and discussion

We demonstrate the use of pairwise inversion signatures for ancestral reconstruction and for phylogenetic reconstruction through extensive simulations. We first show that, under certain reasonable conditions, maximum signatures coincide with ancestral genomes most of the time, then proceed to show that, under more stringent conditions, maximum signatures always coincide with ancestral genomes. Since no polynomial-time algorithm for computing maximum signatures is known at present, we show that our heuristics perform well, both in terms of accuracy and running time, even when applied to larger genomes (to the size of small prokaryotic genome). Finally, we show that the signature method use for phylogenetic reconstruction produces trees comparable in quality to neighbor-joining while providing ancestral reconstructions along the way.

### Maximum signatures as ancestral genomes

Our experiments for ancestral reconstruction simply use triplets of genomes generated from an ancestral genome by generating three evolutionary paths, using randomly chosen inversions. The locations of these inversions is distributed uniformly at random, but their lengths are distributed according to one of two possible distributions: uniform and normal. The lengths of the edges from the ancestor to the three leaves are chosen in both a balanced manner and several skewed manners. All of our experiments used 1,000 repetitions unless stated otherwise and the results presented show averages over these 1,000 tests.

We present most of our results in the form of tables. Tables 1 through 6 group columns by the percentage of the length of the longest simulated path *P *in the triplet. For instance, column two of Table 1 shows the percentage of true ancestors that are within 0 15 × |*P*| inversions away from a maximum signature (in this case, no more than one inversion away because |*P*| is no greater than 8 for any row of column two). The rows in these cases are labeled by the edge length as a percentage of the genome size.

**Table 1:** Percentage of the time that the true ancestor is a maximum signature, under normally distributed inversion lengths on genomes of size *n *= 30.

	% of |*P*|			
# of ops as % of *n*	0	≤ 15%	≤ 20%	≤ 50%
10	97	97	97	100
15	93	93	93	100
20	84	84	93	100
25	78	88	88	100
29	68	83	93	100

**Table 2:** Percentage of the time that the true ancestor is a maximum signature, under uniformly distributed inversion lengths on genomes of size *n *30.

	% of |*P*|			
# of ops as % of *n*	0	≤ 15%	≤ 20%	≤ 50%
10	94	94	94	99
15	87	87	87	100
20	69	69	84	100
25	53	73	73	100
29	36	58	77	100

**Table 3:** Percentage of the time that the true ancestor is a maximum signature as a function of the genome size *n *for simulated edge lengths of *n *× 0 1.

	% of |*P*|			
*n*	0	≤ 15%	≤ 20%	≤ 50%
30	97	97	97	100
35	96	96	96	100
40	95	95	95	100
45	95	95	95	100
50	94	94	98	100
55	95	95	98	100
60	91	91	97	100
65	93	93	98	100
70	91	96	96	100
75	86	92	92	100

**Table 4:** Percentage of the time the true ancestor is a maximum signature, under normally distributed inversion lengths on genomes of size *n *= 100.

	% of *|P*|					
# of ops as % of *n*	0	≤ 5%	≤ 10%	≤ 15%	≤ 20%	≤ 50%
5	95	95	95	95	99	100
8	91	91	91	97	99	100
10	90	90	100	100	100	100

**Table 5:** Percentage of the time that the true ancestor is a maximum (method 1) or maximal (methods 2 and 3) signature, under normally distributed inversion lengths on genomes of size *n *= 30. Method 1 finds a maximum signature by exhaustive search; method 2 uses the greedy Bourque-like approach; and method 3 uses the approach based on maximum sets of noninterfering inversions.

	% of *|P*|				
# of ops as % of *n*	Method	0	≤ 15%	≤ 20%	≤ 50%
	1	97	97	97	100
10	2	97	97	97	100
	3	96	96	96	99

	1	93	93	93	100
15	2	93	93	93	100
	3	89	89	89	100

	1	84	84	93	100
20	2	83	83	92	100
	3	76	76	85	100

	1	78	88	88	100
25	2	76	86	86	100
	3	67	77	77	100

	1	68	83	93	100
29	2	66	81	89	100
	3	57	69	76	100

**Table 6:** Percentage of the time that the true ancestor is a maximal signature, under normally distributed inversion lengths on genomes of size *n *= 30. Method 2 uses the greedy Bourque-like approach while method 3 uses the approach based on maximum sets of noninterfering inversions.

		% of *|P*|					
# of ops as % of *n*	Method	0	≤ 5%	≤ 10%	≤ 15%	≤ 20%	≤ 50%
5	2	95	95	95	95	99	100
	3	94	94	94	94	98	100

8	2	90	90	90	97	99	100
	3	86	86	86	92	94	100

10	2	85	85	94	97	100	100
	3	77	77	85	87	98	100

15	2	68	68	92	98	100	100
	3	54	54	73	90	98	100

20	2	43	63	89	98	100	100
	3	28	41	74	90	98	100

The first set of tables apply to triplets where all edges have the same length (that is, the same number of random inversions). Table 1, for normally distributed inversion lengths, shows that the simulated ancestor is a maximum signature most of the time, even when the evolutionary rates are extremely high. When the rates are already high 10% of the genome size, 97% of the true ancestral genomes are maximum signatures. The table also shows that (the last two rows aside) the true ancestor is within 2 inversions from a maximum signature more than 90% of the time. Table 2 shows similar, but slightly weaker results for uniformly distributed inversion lengths.

The next set of tables examines the influence of the size of the genome. Table 3 shows that the accuracy scales well.

In addition, we tested genomes of size 100; the results are shown in Table 4.

### Computing maximal signatures

The exhaustive algorithm rapidly reaches its limits: for genomes of size 100 with edge lengths of 10, computations already take on the order of hours instead of minutes. Table 4 shows favorable results for exhaustive computation of maximum signatures on such genomes. We now proceed to compare these results with those of our new maximal signature algorithms. Under most circumstances, the true ancestor is found by such maximal signature computations. Table 5 shows that the Bourque-like approach and the approach based on noninterfering inversions fare well with respect to the exhaustive search, the latter dropping off first. Table 6 shows results for the two greedy methods on genomes of size 100. For reasonable rates of evolution (10% or less per edge), we again see that the true ancestor is found most of the time.

Finally, we tested on genomes of more realistic sizes, but of a size usually considered forbidding for ancestral inference–up to 1,000 genes. With 50 random events per edge the Bourque-like computations take just under 30 minutes, while for 80 random events they take under 2 hours. The accuracy remains very high: in 99% of the 380 trials with 50 random events per edge, the signature returned is within 5 inversions of the true ancestor, while in 66% of these trials, the signature returned is in fact the true ancestor. The approach based on noninterfering inversions is by far the fastest, taking under a half a minute for each of these trials, even with 80 random events per edge. Using 50 random inversions per edge, we found that 97% of the 1000 trials gave an ancestor within 5 inversions of the true ancestor, while 57% gave the true ancestor. With 80 events per edge, 91% gave an ancestor within 8 inversions of the true ancestor, while 15% gave the true ancestor.

The largest genomes we tested had size 2000 (corresponding to small bacterial genomes, for instance) and 100 operations per edge, and 5000 (corresponding to the genomes of free-living bacteria such as E. coli) with 250 operations per edge. All trials gave a signature within 10 inversions of the true ancestor, while 90% gave one within 4 inversions, all running in under 2 minutes per trial for size 2000 and 4 minutes per trial for size 5000. These speeds are enormously higher than methods such as MGR or median-based reconstructions, yet the accuracy is also much higher. Thus, by focusing on the characteristic (shared) patterns of inversions, we are able to win on two fronts at once, mostly because we avoid the confusion and long explorations associated with multiple reconverging paths.

### Skewed trees

The true ancestor will not always be equidistant from the leaves and the locus. While large amounts of skew can sometimes move an ancestor farther from a maximum signature, the true ancestor usually remains very close to a maximum signature.

We call the number of random inversions from the locus to the true ancestor *c *and the number of random inversions from the true ancestor to each of the leaves *a *and *b*. We fix *a *to be 10% of the total length and vary *c *and *b *from values equal to *a *up to 2.5 times *a*. Table 7 shows that, for genomes of size 50, the true ancestor is a maximum signature in most cases and that almost as often it is a maximal signature found by the Bourque-like greedy method. Our maximum signature method appears slightly more robust to skew on one of the child branches as opposed to skew on the branch to the locus.

**Table 7:** Percentage of the time that the true ancestor is a maximal signature, under normally distributed inversion lengths on genomes of size *n *= 50. Edge lengths *b *(to a child) and *c *(to an ancestor) vary from 5 to 2*a *while *a *= 5 (number of inversions to the other child). Each entry shows the exhaustive method to the left of the Bourque-like method.

	*c*							
*b*	5		7		10		12	
5	94	94	92	91	87	87	-	82
7	90	90	88	88	82	82	-	79
10	88	88	84	83	80	80	-	73
12	86	86	83	83	-	76	-	66

### Tree reconstruction

**Figure 4:**
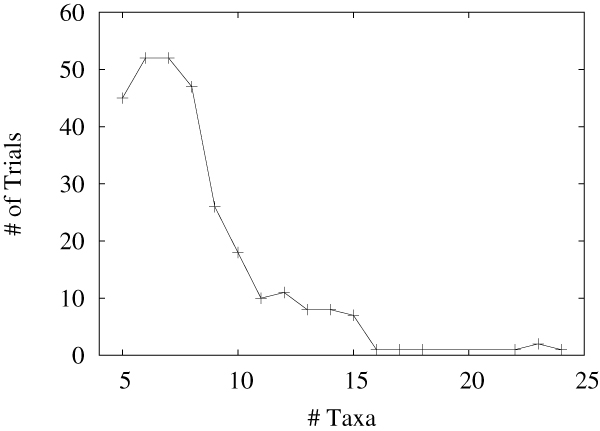
The size of the generated trees.

We simulated evolution over 300 trees to test our signature-based tree reconstruction method. We found that our method (using the Bourque-like signatures for efficiency) reconstructs the true topology most of the time and that any error remains very small. The trees were constructed using the birth-death model and the mean of the normally distributed edge lengths was varied from 5 to 9 operations with a standard deviation varying from 2 to 3. The mean of the normally distributed inversion lengths was varied from 8 to 30 with a standard deviations varying from 5 to 10. The generated trees have from 5 to 24 taxa and are distributed as shown in Figure 4.

Two methods were used for choosing a locus. The first method used the true root of the tree given by the simulation (an ideal method not available in practice, of course), while the second method used a random leaf as the locus. With the true root as the locus, we found that 94% of the trees were reconstructed perfectly, while 16 of the 17 remaining trees had a Robinson-Foulds error of 2, giving an average RF error of 0.15. With a random leaf as the locus, we found that 85% of the trees were reconstructed perfectly, while 28 of the 45 remaining trees had an RF error of 2 and 11 of the last 27 had an RF error of 4, giving an average RF error of 0.5.

Using the true root as the locus demonstrates that the pairwise signature contain a great deal of information about the phylogeny. Using a random leaf as the locus demonstrates that such information remains recoverable even when the choice of locus is arbitrary (and usually far from ideal), justifying our initial claim that comparing two genomes with respect to a third tremendously enriches what can be had from a direct pairwise comparison. (As an example, trees that were not properly reconstructed by the neighbor-joining method, which uses strictly pairwise comparisons, were commonly reconstructed correctly by our signature-based method.) Our tests for phylogenetic reconstruction are obviously of limited scope, meant to exemplify the usefulness of the method rather than provide a full evaluation; and the method itself is subject to many obvious improvements (better ways to choose a locus, using *k*-way signatures rather than pairwise ones to support a top-down reconstruction method, etc.)

### Tightness of the upper bound

Finally, we present experimental results suggesting that our upper bound is on average very tight and then use the bound to show that the greedy signatures, used for ancestral reconstruction of genomes too large for the exhaustive computation, are indeed close to a maximum signature. Since the computed ancestor is bracketed within this bound, our results imply that the maximum signature is very close to the true ancestor with high probability.

The upper bound was computed for each trial in Table 1. For each of the sets of 1000 trials, the average difference between the upper bound and the maximum signature was 0.029, 0.073, 0.176, 0.27, and 0.327 for trials with 10, 15, 20, 25, and 29 percent respectively. For the length-dependent data from Table 3, the average difference stays between 0.021 and 0.082. Table 8 indicates similar performance for experiments run on skewed triplets. The tests from Table 6 give average differences from 0.024 up to 1.375 for the Bourque-like method and differences from 0.048 up to 2.228 for the noninterfering inversions method. Only one of the tests from genomes of size 1000 did not match the upper bound for the greedy method.

**Table 8:** The average difference between the upper bound and the computed signatures with normally distributed inversion lengths and genomes of size *n *= 50. Edge lengths *b *(to a child) and *c *(to an ancestor) vary from 5 to 2*a *while *a *= 5 (number of inversions to the other child). Each entry shows the exhaustive method to the left of the Bourque-like method.

	*c*							
b	5		7		10		12	
5	0.053	0.053	0.080	0.081	0.138	0.143	-	0.176
7	0.106	0.106	0.114	0.114	0.173	0.173	-	0.224
10	0.097	0.098	0.165	0.167	0.203	0.203	-	0.290
12	0.131	0.132	0.158	0.158	-	0.279	-	0.359

## Conclusion

In any study of evolutionary changes, the challenge is to distinguish global patterns from a background of many local changes–or, to put it another way, to find commonalities among many equally plausible evolutionary paths that lead to the same modern organism. We have proposed an approach to this problem that focuses on intermediate states along such paths in the setting of a speciation event and seeks to return the last (most recent) states from which both species of organisms could still have been derived. This approach offers multiple benefits: the focus on intermediate states translates readily into one on ancestral reconstruction; the study of paths going through a fork (the speciation event) stresses the role of evolutionary history rather than just final states; and the search for the most recent states that are part of the fork naturally separates common evolutionary changes (prior to the fork) from individual variations (subsequent to the fork). Although finding such signatures appears hard, we gave an efficient heuristic that does very well through an extensive range of simulations. Our signatures are based on inversions, since inversions are the best studied of the various genomic rearrangements to date, but the concept readily extends to any other rearrangement operation or family of such operations.

## Competing interests

The authors declare that they have no competing interests.
